# HSF1 induces RNA polymerase II synthesis of ribosomal RNA in *S. cerevisiae* during nitrogen deprivation

**DOI:** 10.1007/s00294-021-01197-w

**Published:** 2021-08-06

**Authors:** Arjuna Rao Vallabhaneni, Merita Kabashi, Matt Haymowicz, Kushal Bhatt, Violet Wayman, Shazia Ahmed, Heather Conrad-Webb

**Affiliations:** 1 Department of Biology, Texas Woman’s University, 304 Administration Dr., Denton, TX 76204 USA; 2 Department of Bioinformatics, University of Texas Southwestern, 5323 Harry Hines Blvd., Dallas, Texas 75390 USA

**Keywords:** rRNA synthesis, Polymerase switch, Hsf1, Nitrogen deprivation

## Abstract

The resource intensive process of accurate ribosome synthesis is essential for cell viability in all organisms. Ribosome synthesis regulation centers on RNA polymerase I (pol I) transcription of a 35S rRNA precursor that is processed into the mature 18S, 5.8S and 25S rRNAs. During nutrient deprivation or stress, pol I synthesis of rRNA is dramatically reduced. Conversely, chronic stress such as mitochondrial dysfunction induces RNA polymerase II (pol II) to transcribe functional rRNA using an evolutionarily conserved cryptic pol II rDNA promoter suggesting a universal phenomenon. However, this polymerase switches and its role in regulation of rRNA synthesis remain unclear. In this paper, we demonstrate that extended nitrogen deprivation induces the polymerase switch via components of the environmental stress response. We further show that the switch is repressed by Sch9 and activated by the stress kinase Rim15. Like stress-induced genes, the switch requires not only pol II transcription machinery, including the mediator, but also requires the HDAC, Rpd3 and stress transcription factor Hsf1. The current work shows that the constitutive allele, Hsf1^PO4*^ displays elevated levels of induction in non-stress conditions while binding to a conserved site in the pol II rDNA promoter upstream of the pol I promoter. Whether the polymerase switch serves to provide rRNA when pol I transcription is inhibited or fine-tunes pol I initiation via RNA interactions is yet to be determined. Identifying the underlying mechanism for this evolutionary conserved phenomenon will help understand the mechanism of pol II rRNA synthesis and its role in stress adaptation.

## Introduction

Ribosomal synthesis is the most resource intensive cellular process, and cell viability is highly dependent on the precise production and assembly of ribosomes. Thus, ribosome production is tightly linked to growth rate and the need for protein synthesis (Kief and Warner 1981) with RNA polymerase I (pol I) transcription of rRNA being the rate limiting step (Laferté et al. 2006; Chedin et al. 2007). During nutrient deprivation in yeast, inhibition of the TOR and PKA signaling pathways greatly prohibits pol I pre-initiation complex (PIC) formation reducing pol I transcription to ~ 10% of maximal transcription (Warner 1999; Fahy et al. 2005). In addition to nutrient depletion, stress conditions result in a rapid inhibition of pol I transcription (Warner 1999; Grummt 2010). In this paper, we demonstrate that the processes that prohibit pol I PIC formation also triggers Heat Shock Transcription Factor 1 (Hsf1) mediated synthesis of rRNA by RNA polymerase II (pol II). This polymerase switch employs factors required for general stress response in yeast classifying pol II rRNA synthesis as a stress response. Furthermore, the synthesis of rRNA by pol II is evolutionarily conserved suggesting that it contributes to the regulation of rRNA synthesis, in general.

During normal growth in response to TOR and PKA signaling, the pol I transcription factor UAF binds to the upstream element of the pol I rDNA promoter allowing the further binding of the Core Factor, TBP and Rrn3/pol I to the core promoter. (Fath et al. 2001; Moss et al. 2007; Woolford and Baserga 2013). Astonishingly, yeast cells survive in the absence of UAF and the ensuing inhibition of most, if not all, pol I transcription. Absence of UAF subunits Rrn5, Rrn9 or Rrn10 result in the loss of UAF binding and pol I transcription with a subsequent reduction in growth rate. The absence of UAF binding results in dramatic changes in rDNA promoter chromatin and the exposure of an overlapping cryptic pol II rDNA promoter (Goetze et al. 2010) allowing these strains to survive by the induction of a switch to pol II synthesis of the 35S rRNA (Keys et al. 1996; Oakes et al. 1999; Vu et al. 1999; Siddiqi et al. 2001).

**Fig. 1:**
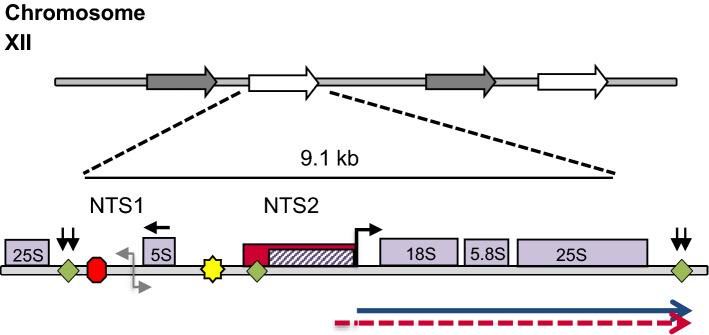
The overlapping pol I and pol II rDNA promoters within the rDNA repeat. In *S. cerevisiae*, 100–200 copies of the rDNA repeat are clustered on chromosome XII; however, only approximately half are available or “open” for transcription. Synthesis of the 35S rRNA by pol I is dependent upon the pol I promoter (hatched box), which contains the binding sites for UAF and the core factor, CF. The overlapping pol II rDNA promoter (solid box) is used in rho^0^ cells or stressed cells to synthesize a 35S rRNA (dashed arrow), while wildtype rho^+^ cells or non-stressed cells use the pol I promoter to synthesize the 35S rRNA (arrow). Reb1 binding sites (diamond), Replication Fork Barrier (RFB, octagon), Enhancer promoter (E-pro, gray arrow) and the ARS (star) are also shown. Direction of 35S rRNA transcription is shown as a bent arrow. 35S rRNA termination sites are shown as downward arrows

This polymerase switch also occurs in wildtype yeast experiencing mitochondrial dysfunction. Rho^0^ cells that lack mitochondrial DNA, and therefore are unable to respire, transcribe a significant percentage of the 35S ribosomal RNA precursor using pol II. Pol II synthesized rRNAs are incorporated into ribosomes and able to support growth in the absence of the pol I subunit RPA135 and functional pol I (Conrad-Webb and Butow 1995) (Fig. 1). Likewise, Nomura’s laboratory observed pol II rRNA transcription in wildtype strains when grown at 37 °C (Oakes et al. 1999). Like the polymerase switch induced in UAF deletion strains, the environmentally induced polymerase switch requires both transcriptional activation and extensive chromatin remodeling. Intriguingly, pol II synthesis of rRNA is observed in higher eukaryotes—including mammalian systems—when the binding of the pol I species specific transcription factor, SL1, is impaired. (Dhar et al. 1985, 1987; Smale and Tjian 1985). Similarly, cryptic pol II rDNA promoters have been discovered in plants (Doelling and Pikaard 1996).

The ability of pol II to transcribe rRNA in such diverse organisms as yeast, plants and mammalian cells strongly supports the hypothesis that the polymerase switch is a universal phenomenon. In higher eukaryotes, the switch has been observed when SL1 is inhibited from binding to the rDNA promoter. In yeast, the polymerase switch occurs during chronic heat stress or mitochondrial dysfunction when stress pathways are activated and growth signaling pathways inhibit assembly of the pol I transcription pre-initiation complex (and UAF binding). Even during stationary phase or quiescence, rRNA and ribosome continue to be synthesized although at greatly reduced levels allowing cell to adapt and survive severe nutrient deprivation (Fahy et al. 2005; Bierhoff et al. 2014; Roche et al. 2017). Thus, pol II synthesis of rRNA may serve as a backup mechanism for rRNA synthesis during stress conditions allowing fine-tuning of rRNA synthesis and the survival of the cell.

In this paper, we investigate the regulation of the polymerase switch and demonstrate that nitrogen deprivation also induces the switch. Similar to stress response genes in yeast, the induction of the switch is repressed by growth signaling kinase Sch9 and is activated by the stress kinase Rim15. Like stress-induced genes, the switch requires not only pol II transcription machinery including the mediator, but also requires the HDAC, Rpd3. Finally, Heat Shock Factor 1 modulates pol II transcription of rRNA suggesting that the polymerase switch occurs in response to chronic stress.

## Methods and materials

### Yeast strains, plasmids and general methods

Yeast strains used in this study are as listed in Table 1. All strains from the Saccharomyces Genome Deletion Project (Winzeler et al. 1999) were purchased from Open Biosytems. TAP-tagged strains (Ghaemmaghami et al. 2003) were purchased from Dharmacon. The rDNA-lacZ reporter plasmid, pFES17, is a derivative of YEprlacZ-1208 (Conrad-Webb and Butow 1995) where the reporter gene was transferred into Yep366 (Myers et al. 1986) to allow for leucine selection. pYW2A4Δ is a derivative of YIprT7*λ* (Conrad-Webb and Butow 1995) with a shortened T7 reporter. Designated strains were transformed using the Gietz Transformation Method (Gietz and Woods 2002). All integrations, deletions and TAP fusion strains were confirmed by PCR. Oligonucleotides used for copy number determination and ChIP assays are listed in Table 2.

**Table 1:** Yeast Strains used in this study

Strain name	Genotype	Source
PSY142 rho^+^/rho^0^	*MATα leu2 lys2 ura3 rho*^+^*/rho*^*0*^	R. A. Butow
*rtg1∆ rho*^+^*/rho*^*0*^	PSY142 *rtg1::URA3*	R. A. Butow
*rtg2∆ rho*^+^*/rho*^*0*^	PSY142 *rtg2::LEU2*	R. A. Butow
*rtg3∆ rho*^+^*/rho*^*0*^	PSY142 *rtg3:: LEU2*	R. A. Butow
*rtg1∆ rtg3∆ rho*^+^*/rho*^*0*^	PSY142 *rtg1::URA3 rtg3:: LEU2*	R. A. Butow
BY4741	*MATa his3Δ1 leu2Δ0 met15Δ0 ura3Δ0 pFES17*	R. A. Butow
BY4741-T7	*BY4741 rDNA::* pYW2A4Δ *pFES17*	This study
Reb1-TAP	*BY4741 REB1::REB1-TAP*	Dharmacon
Reb1-TAP T	BY4741 Reb1-TAP *rDNA::* pYW2A4Δ *pFES17*	Dharmacon
Gis1-TAP T7	BY4741 Gis1-TAP *rDNA::* pYW2A4Δ *pFES17*	Dharmacon
BY4743 wildtype	*MATa/α his3Δ1/his3Δ1 leu2Δ0/leu2Δ0 met15Δ0/MET15 LYS2/lys2Δ0 ura3Δ0/ura3Δ0 pFES17*	This study
*gal11∆*	BY4743 *gal11:: KanMAX pFES17*	This study
*pdg1∆*	*BY4743 pdg1:: KanMAX pFES17*	This study
*Ssn8∆*	*BY4743 ssn8:: KanMAX pFES17*	This study
*rpd3∆*	*BY4741 rpd3::KanMAX pFES17*	This study
*sin3∆*	*BY4741 sin3::KanMAX pFES17*	This study
*dep1∆*	*BY4741 dep1::KanMAX pFES17*	This study
*rco1∆*	*BY4741 rco1::KanMAX pFES17*	This study
*kcs1∆*	*BY4741 kcs1::KanMAX pFES17*	This study
*vip1∆*	*BY4741 vip1::KanMAX pFES17*	This study
*tor1∆*	*BY4743 tor1::KanMAX pFES17*	This study
*sch9∆*	*BY4743 sch9::KanMAX pFES17*	This study
*rim15∆*	*BY4743 rim15::KanMAX pFES17*	This study
*gis1∆*	*BY4743 gis1::KanMAX pFES17*	This study
*msn2∆*	*BY4743 msn2::KanMAX pFES17*	This study
*msn4∆*	*BY4743 msn4::KanMAX pFES17*	This study
AGY249	*Mat a his3Δ1 leu2Δ0 met15Δ0 ura3Δ0 msn2::KANMX msn4::URA3*	*A. Gasch*
WT	*W3031a MATa ADE2 HSE-YFP::LEU2*	*D. Pincus*
DPY416	W303 *MATa hsf1∆::KAN 4xHSE-YFP::LEU2 HSF1pr-HSF1∆po4::TRP1*	*D. Pincus*
DPY605	W303 MATa hsf1∆::KAN 4xHSE-YFP::LEU2 HSF1pr-HSF1PO4*::TRP1	*D. Pincus*

**Table 2:** Primers used in this study

Target	Forward	Reverse
*AmpR*	5' CTGCATAATTCTCTTACTGTCATGCC	5' GTATTGACGCCGGGCAAGAGC
*TRP1*	5' GTTCAGGCACTCCGAAATACTTGG	5' GGAACTCTTGGTATTCTTGCCACG
*rDNA 1*	5' TCCGTTGGTTTTGGTTTCGGT	5' ATTTTCTGCCCTCTCTGTCG
*T7*	5' TCCGTTGGTTTTGGTTTCGGT	5' TACAACGTGTGGAACTTCGG
*LacZ*	5' TCCGTTGGTTTTGGTTTCGGT	5' CAGTCACGACGTTGTAAACGAC
*CTT1*	5' TTTCGACGTTGAAGATTAGGGG	5' CGCAATTTCACCGCTTGG
*HSP26*	5' ACTCCGTGTGTACCCCTAACTC	5' TTTGGACGCATAAGGGGGA
*TRA1*	5' ATCAAACAATCCGCGTACCTGGTTAC	5' GCGTTCATACGTTCATGGAGAACAGG

### Growth conditions

Strains transformed with pFES17 were grown in synthetic complete media lacking leucine (SC) (0.67% yeast nitrogen base, 1% ammonium sulfate, 2% dextrose and 0.2% of SC-leu dropout synthetic mix without leucine and nitrogen base, US Biological) at 30 °C with shaking. For nitrogen rich conditions (SC), cells were harvested at OD_600_ = 0.8–1.0. For nitrogen deprivation, cells were initially grown in SC broth until OD_600_ ~ 0.35. Cells were collected by centrifugation, washed twice with sterile distilled water and resuspended in low nitrogen SLAD media (LN)(0.17% yeast nitrogen base without amino acids, 2% glucose and 0.025% ammonium sulfate and 20 µg/L of uracil and histidine) and incubated with shaking at 30 °C for 12 h (Gasch et al. 2000).

### β-Galactosidase assays

β-Galactosidase activity was measured in whole cell extracts as previously described in three independent experiments (Rose and Botstein 1983). In each experiment, at least two extracts were assayed in triplicate. β-Galactosidase activity was corrected for relative plasmid copy number by quantitative real time PCR measured in quadruplets. Relative copy number was calculated by the ^∆∆^Ct method (Livak and Schmittgen 2001) comparing the average Ct value for plasmid *ampR* gene to the genomic *TRP1* gene. The plasmid copy number was normalized to wildtype copy number (1.0) in normal nitrogen conditions. Data (*n* ≥ 3) was analyzed using a One-Way ANOVA and Tukey HSD Post Hoc test, *p* < 0.05.

### Chromatin immunoprecipitation (ChIP) assay

ChIP experiments were performed using Tandem Affinity Purification (TAP) tagged strains (Ghaemmaghami et al. 2003) or wildtype BY4741 strains containingYW2A4Δ integrated into the rDNA repeat and the multicopy pFES17 plasmid. Non-TAP-tagged strains as well as IGG were used as negative controls. In at least three independent experiments, strains were grown in normal (SC) and low nitrogen media (LN) as described previously. The ChIP protocol was adapted from Kurdistani and Grunstein (2003). Briefly, Cells were crosslinked initially with 10 mM dimethyl adipimate (DMA) in ice-cold PBS with 0.25% DMSO for 45 min. The cells were harvested and washed in ice-cold PBS, followed by crosslinking in 1% formaldehyde for 1 h. The crosslinking was terminated by addition of 2.5 M glycine. The cell pellet was resuspended in 1 ml of freshly prepared ChIP lysis buffer (50 mM HEPES–KOH pH 7.5, 500 mM NaCl, 1 mM EDTA, 1% Triton X-100, 0.1% Sodium Deoxycholate, 0.1% Sodium Dodecyl Sulfate) with protease inhibitor cocktail (AEBSF Sigma # S8830) and 1 mM PMSF. Cells were lysed using 0.5 mm glass beads in a Mini-Beadbeater™ (Biospec) for a total of 5 min in 1 min intervals, followed by sonication using a Qsonica sonicator for 3 min at 100% amplitude with 20 s ON and 40 s OFF cycles to obtain DNA fragments ranging from 0.4 to 1 kb. The whole cell extract was clarified by centrifugation and protein estimation was performed using BCA assay kit (Thermoscientific Cat# 23250). Immunoprecipitation reactions were set up overnight using 400 µg of protein and the appropriate antibody, reserving 1% for input controls. Antibodies used were rabbit anti-TAP antibody (Thermoscientific # CAB1001), rabbit anti-H3 antibody (Cell Signaling #4620), rabbit anti-Msn4 (Santa Cruz sc-15550), rabbit anti-pol II and rabbit anti-Hsf1 (gifts of D. Gross). Complexes were isolated with Protein A/G agarose beads or G magnetic beads and the eluted protein-DNA complexes were digested. DNA was isolated using a PCR purification kit (Qiagen QIAquick PCR purification kit # 28,104). Quantification of DNA was performed using real time PCR (Bio-Rad # 1,725,274) with respective primer sets. Percent input [100*2^(Adjusted input—Ct (IP)] was calculated and analyzed using a two-tailed Student’s Independent *t* test at *p* < 0.05 (*n* ≥ 3).

## Results

To investigate the hypothesis that pol II synthesizes rRNA during stress conditions other than mitochondrial dysfunction, we inspected the pol II rDNA promoter for putative transcription factor binding sites using YeTFaSCo (De Boer and Hughes 2011). Previous investigations had established that the pol II rDNA promoter overlapped the pol I promoter extending upstream of the Reb1 binding site (Conrad-Webb and Butow 1995) (Fig. 1). The pol II rDNA promoter (− 1 to − 380) contains numerous putative transcription factor binding sites, many of which are activated during nutrient limitation, stress response and adaptation (Cherry et al. 2012). Three putative transcription factor sites were of great interest: the retrograde transcription heterodimer Rtg1/Rtg3, Heat Shock Factor Hsf1, and the general stress response factors, Msn2/Msn4 (Table 3).

**Table 3:** Transcription factors with putative sites within the rDNA pol II promoter and their functions^a^

Oxidative stress	Chromatin remodeler required for stress response	Nutrient limitation	Other stresses multiple stress	Repressor of Stress response genes
Yap1 (5)	Rsc3 (3)	Gis1	Pdr3 (5)	Stb3
Yap5 (2)	Rsc30	Rph1 (2)	Sut1 (2)	Arg81 (2)
Yap6		Put3 (3)	Mot3 (2)	Mig1
Skn7 (2)		Pho4	Hal9 (3)	Mig2
		Phd1	Asg1 (5)	Rdr1 (5)
		Sip4	Out1 (5)	
		Gal4	Fkh2 (2)	
		Aro80 (4)	Uvs1	
		Gcr1 (2)	Msn2 (2)	
		Mga1 (2)	Msn4 (2)	
		Leu3 (2)	Haa1 (2)	
		Rgm1 (2)	Hsf1 (2)	
		Azf1	Rtg1/3	

^a^Numbers in parenthesizes are number of putative binding sites

**Fig. 2:**
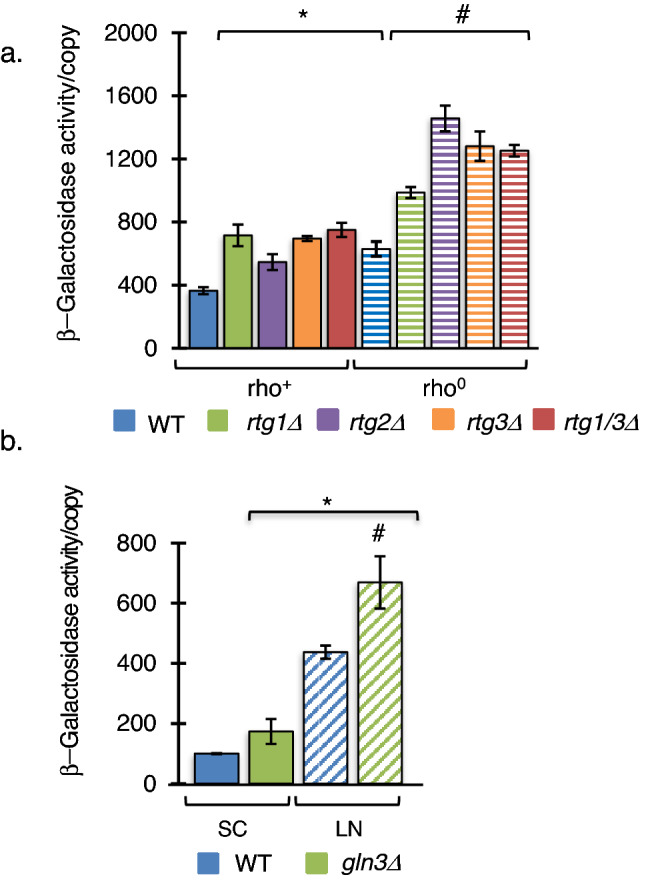
Defects in the retrograde pathway or nitrogen utilization induce Pol II rRNA synthesis. **a** Rho^+^ and rho^0^ derivatives of wildtype PSY142, *rtg1∆*,* rtg2Δ, rtg3Δ* or double-deletion *rtg1Δ rtg3Δ* strains containing the reporter plasmid YEprlacZ-1208 were grown on SC Raffinose medium and assayed for β-galactosidase activity/copy from whole cell extracts. *Significant compared to wildtype rho^+^ cells, #Significant compared to wildtype rho^+^ and rho^0^ cells. (One-Way ANOVA, Tukey’s HSD, *p* ≤ 0.005, *n* = 3). **b** Wildtype BY4741 rho + and *gln3∆* strains containing the pFES17 reporter plasmid were grown on selective media (SC) or low nitrogen medium (LN) for 12 h. Error bars represent standard error of the mean (SEM) *significant compared to wildtype rho^+^ cells grown in normal nitrogen (SC), #significant compared to wildtype rho^+^ cells in either condition (One-Way ANOVA, Tukey’s HSD, *p* ≤ 0.01, *n* = 3)

The polymerase switch and retrograde response are intimately linked. First, their discovery came from the same investigation on the influence of the mitochondrial genome quality on nuclear gene expression (Parikh et al. 1987, 1989; Liu and Butow 1999). Second, both the retrograde response and pol II synthesis of rRNA are highly induced in rho^0^ cells, but not in rho^+^ cells. Finally, the putative Rtg1/Rtg3 binding sites exist within the pol II rDNA promoter. To test the role of the retrograde pathway in pol II rRNA synthesis, wildtype PSY142 rho^+^ or rho^0^ strains and *RTG* deletion derivatives harboring a rDNA-*lacZ* reporter plasmid were examined for β-galactosidase activity/reporter gene copy when grown on SC-Raffinose media (Fig. 2a). Although both pol I and pol II RNAs are produced from the reporter gene, only pol II RNAs are translated (Lopata et al. 1986), thus β-galactosidase activity is a measure of pol II mediated rRNA synthesis. In contrast to our hypothesis that *rtg1∆*, *rtg2∆*, *rtg3∆* and double-deletion *rtg1∆*, *rtg 3∆*, strains should have decreased reporter gene activity, the absence of Rtg1, Rtg2, or Rtg3 resulted in a significant increase in β-galactosidase activity in rho^+^ cells, an increase comparable to the increase in wildtype rho^0^ cells. In addition, reporter gene activity in *rtg1∆, rtg2∆*, *rtg3∆* and *rtg1∆/rtg 3∆* rho^0^ cells was further increased compared to wildtype rho^0^ cells. Since mitochondrial dysfunction activates both the retrograde pathway and the polymerase switch, the absence of the Rtg1/Rtg3 transcription factors must activate the polymerase switch indirectly. *rtg1∆, rtg2∆* or *rtg3∆* strains show glutamate autotrophy (Dilova and Powers 2006) indicating that the glutamate is limiting in the absence of Rtg1, Rtg2 or Rtg3. We, therefore, hypothesized that nitrogen deprivation may induce the polymerase switch.

### Nitrogen deprivation induces the polymerase switch

To test the hypothesis that pol II rRNA synthesis occurs during nitrogen deprivation, we tested wildtype cells and cells lacking Gln3 transcription factor, an activator of nitrogen catabolic repression (NCR) genes during nitrogen deprivation and another gluatamate auxotroph. Wildtype BY4743 rho^+^ or *gln3∆* cells harboring pFES17 were transferred during early log phase to nitrogen deprivation media (LN) and grown an additional 12 h. Alternatively, cells were grown in synthetic complete (SC) media until mid-log phase. Nitrogen deprived wildtype cells showed an approximately fourfold increase in β-galactosidase activity, which confirms that nitrogen deprivation does induce the polymerase switch (Fig. 2b). Furthermore, *gln3∆* cells showed β-galactosidase activity elevated over the wildtype controls grown in the same conditions revealing a link between the polymerase switch and nitrogen availability (Fig. 2b).

**Fig. 3:**
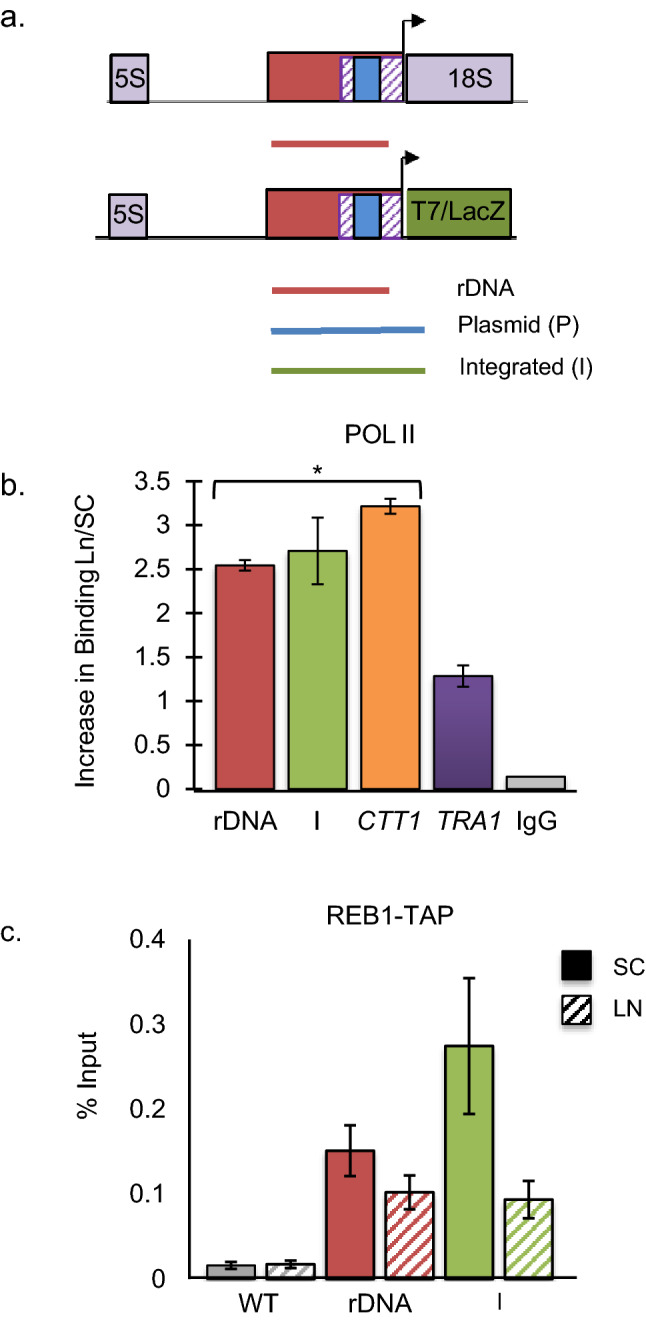
Nitrogen deprivation induces pol II Synthesis of rRNA. **a** The overlapping pol I (striped box) and pol II rDNA promoter (solid red box) of the ribosomal repeat and T7/LacZ reporter genes are shown. Regions amplified for ChIP analysis are shown below. Primer set rDNA (red) amplifies rDNA promoters in all contexts: open and closed rDNA repeats, as well as, the promoters of lacZ or T7 reporter genes (Conrad-Webb and Butow 1995). The plasmid (P, blue) or integrated (I, green) primer sets amplify only their respective promoters. All primer sets have the same 5’ boundary upstream of the pol I promoter. Pol I UAF binding site (solid blue box). **b** Yeast TAP-tagged and untagged strains, with the rDNA-T7 reporter gene integrated into a single rDNA repeat and pFES17 were grown in SC media (solid bar) prior to transfer to SLAD media (LN) for 12 h (striped bar). Harvested cells were subjected to ChIP using anti-pol II (gift of D. Gross) and the purified DNA was analyzed using specific primers (Fig. 3a) by q-PCR. Pol II binding at rDNA promoter is represented as a ratio of pol II binding in LN/pol II binding in SC media. As controls, pol II binding at the *CTT1* promoter (orange) and at an internal coding region of the *TRA1* gene (purple) are shown. Normal IgG antibody (gray) serves as negative control. Error Bars = SEM. *Significant increase in pol II binding (LN/SC) compared to pol II binding to *TRA1*. (Independent Student *T* test, *p* < 0.05, *n* = 3). **c** As a control experiment, Reb1 a protein known to bind to the pol I/pol II rDNA promoter was subjected to ChIP analysis in parallel using the Reb1-TAP-tagged strain and anti-TAP antibody (Thermo fisher). As expected, Reb1 binds to the pol II promoter in all contexts, but did not bind to the *TRA1* or *CTT1* genes. Error Bars = SEM, *n* = 3. Independent Student *T* test, *p* < 0.05

To confirm increased Pol II synthesis of rRNA during nitrogen deprivation, ChIP experiments were conducted to examine pol II binding to the rDNA promoter. In yeast, rDNA exists in a tandem array of ~ 150 rDNA repeats on chromosome XII where ~ 50% of the 150 rDNA chromosomal repeats are accessible for transcription (open repeats) (Merz et al. 2008; Gartenberg and Smith 2016). Thus, the rDNA promoter is present in multiple contexts- chromosomal open repeats, chromosomal closed repeats and extrachromosomal repeat circles (ERCs). Our strain also harbors the reporter plasmid with its own rDNA promoter. To clarify the class of rDNA promoter bound by pol II, a series of unique primer sets were devised (Fig. 3a). Set rDNA amplifies all rDNA promoters and T7 (I) amplifies a single open rDNA repeat with a unique T7 insertion in the 35S transcription unit. ChIP analysis demonstrated that binding to the pol II rDNA promoters in both contexts significantly increased ~ 2.5 fold following nitrogen deprivation (Fig. 3b). Similarly, binding to the *CTT1* promoter, a gene where expression increases ~ 4.5 fold following 12 h of nitrogen deprivation (Gasch et al. 2000), shows a similar increase in pol II binding, while binding to an internal region of the *TRA1* gene shows no change in pol II binding under low nitrogen conditions as is expected for a gene not regulated by nitrogen deprivation. As a positive control, Reb1, a protein known to bind to the promoter and enhancer regions of the rDNA repeat (Morrow et al. 1989), binds to rDNA promoters in all contexts (Fig. 3c).

**Fig. 4:**
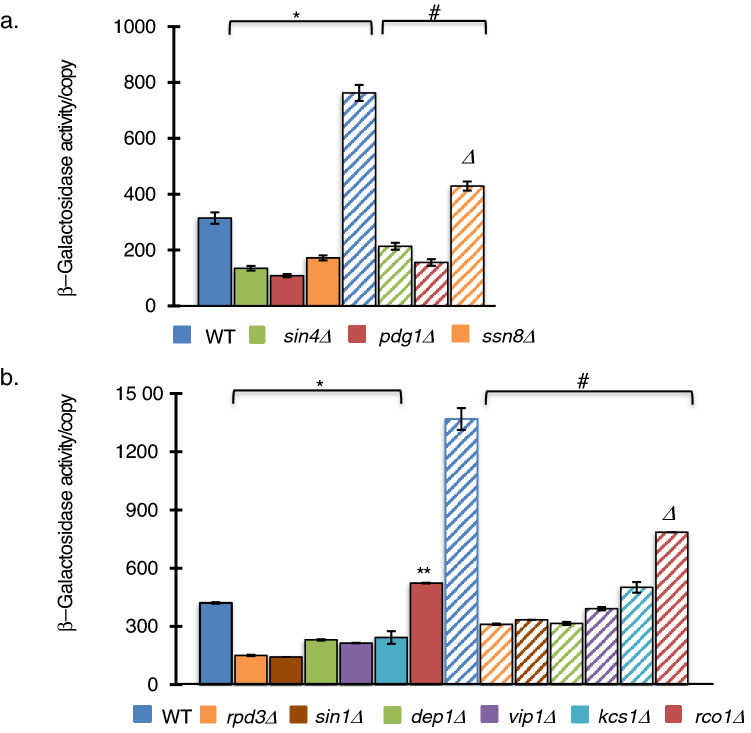
Stress Transcription machinery, the mediator and HDAC Rpd3, are required for the Pol II rRNA synthesis. **a** β-galactosidase activity/copy in wildtype BY4743 and mediator tail deletion strains, *sin4∆* and *pdg1∆*, or CDK module deletion strain *ssn8*∆ were assayed in SC and LN as described above. All strains showed significantly decreased activity from wildtype grown in normal nitrogen (*) or low nitrogen conditions (#). Furthermore, CDK module deletion strain *ssn8*∆ showed a distinct intermediate phenotype in low nitrogen conditions (∆). (One-Way ANOVA, Tukey’s HSD, *p* ≤ 0.001, *n* = 3). **b** Wildtype BY4741 rho^+^ and unique RpdL deletion derivative *dep1∆*, RpdS derivative *rco1∆* and inositol hexaphosphate kinase deletion strains, *vip1∆* and *kcs1∆*, were assayed for β-galactosidase activity/reporter copy number from cells grown in normal (SC) or nitrogen deprivation (LN) conditions. All strains showed significantly different activity from wildtype grown in normal nitrogen. RpdS derivative *rco1∆* cells grown in either SC (**) or LN (∆) formed a unique group, significantly different from all deletion strains grown in the same media. *Significant compared to wildtype BY4741 cells grown in SC. #Significant compared to wildtype BY4741 in LN Error Bars = SEM. (One-Way ANOVA, Tukey’s HSD, *p* ≤ 0.001, *n* = 3)

The highly conserved mediator complex plays a crucial role in the assembly of the Pol II pre-initiation complex linking gene specific transcription factors to the basal RNA polymerase II complex (Paul et al. 2015). The mediator binds to the promoters of stress-induced genes (Paul et al. 2015) and is required for the proper induction of stress responses including the Environmental Stress Response (ESR) (Ansari and Morse 2012). Disruption of interaction between the mediator tail module and stress transcription factors results in aberrant or no stress response (Fan et al. 2006; Ansari and Morse 2012, 2013; Kim and Gross 2013). To determine if the mediator is required for pol II rRNA synthesis during nitrogen deprivation, we assayed deletion strains of the tail module components (*sin4/med16∆; pdg1/med3∆)* and the CDK module (*ssn8∆*) for β-galactosidase activity (Fig. 4a). Even in an abundance of nitrogen, all three mediator deletion strains showed significant reduction in β-galactosidase activity. Following 12 h of nitrogen deprivation, the absence of tail subunits resulted in a further decrease in β-galactosidase activity well below the level of activity of the wildtype strain grown in the presence of nitrogen. The absence of the Ssn8 subunit of the CDK8/CycC module during nitrogen deprivation resulted in a significant, but less dramatic reduction in enzyme activity. Thus, the absence of the mediator significantly impairs both pol II rRNA synthesis as well as the transcription of stress response genes.

A second component required for induction of many stress responsive genes is the HDAC Rpd3 (Sertil et al. 2007; Alejandro-Osorio et al. 2009). The Rdp3 complex exists in three conformations: Rpd3L functions in transcription initiation, Rpd3S plays a role in transcription elongation and the Snt2C complex plays a role in oxidative stress (Baker et al. 2013). In addition, Rpd3L is known to be required for the polymerase switch in strains lacking UAF (Oakes et al. 2006). To determine if Rpd3 is required for the induction of pol II rRNA synthesis during nitrogen deprivation conditions, β-galactosidase activity was measured in deletion strains of the Rpd3 core subunits, as well as unique subunits of the RpdL and RpdS complex (Fig. 4b). Absence of either Rpd3 or Sin3 of the core complex yielded a similar and significant reduction in β-galactosidase activity during non-stress and stress conditions. Absence of the Dep1 subunit of Rpd3L also resulted in a significant decrease in reporter gene activity in both conditions. In contrast, the absence of the Rco1 of the Rpd3S complex resulted in small increase during non-stress conditions and a modest decrease during nitrogen deprivation.

Rpd3L mediated-induction of stress response is dependent upon the binding of the inositol pyrophosphate (PP-IP), since the absence of PP-IP or defects in the PP-IP binding site of Rpd3 lead to the inability of cells to respond to heat, osmotic or oxidative stress (Worley et al. 2013). To investigate the role of PP-IPs in the polymerase switch, we evaluated the effect of the absence of the inositol hexakisphophate kinases (IP6Ks), Vip1 and Kcs1, on reporter gene function. The absence of either kinase resulted in a significantly lower β-galactosidase activity during nitrogen deprivation similar to the levels resulting from the loss of Rpd3L components in *dep1∆*, *rpd3∆* and *sin3∆* strains. Thus, Rpd3L is required for the polymerase switch not only in the absence of pol I transcription factor UAF, but also during environmental conditions of nitrogen deprivation. The requirement of hexokinases Vip1 and Kcs1 suggests that Rpd3 deacetylase activity is also necessary for induction of the switch similar to Rpd3 induction of oxidative, osmotic and heat shock responses (Alejandro-Osorio et al. 2009; Worley et al. 2013).

### Stress signaling pathways regulate pol II rDNA synthesis

**Fig. 5:**
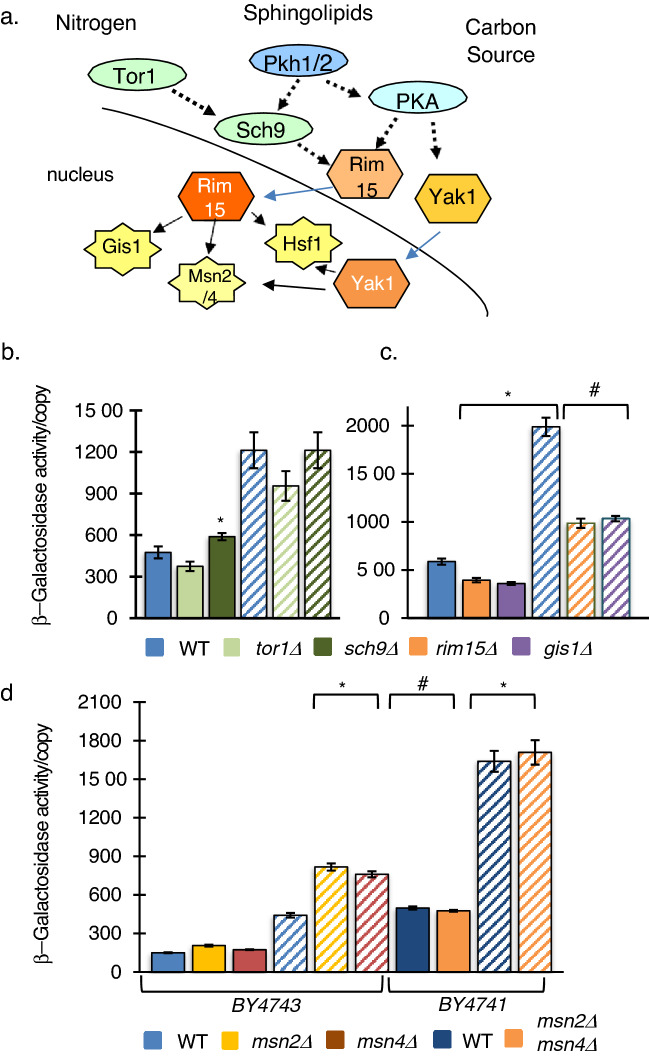
Stress signaling pathways regulate pol II rDNA synthesis. **a** During nutrient limitation, Tor1, Sch9, PKA, and Pkh1/2 kinases no longer activate growth pathways or inhibit stress pathways (dashed arrow). Activated stress kinases Rim15 and Yak1 translocate to the nucleus where they activate (solid arrow) downstream transcription factors Msn2/4, Gis1 and Hsf1. **b** Wildtype BY4743, *tor1Δ* and *sch9Δ* strains or **c** *rim15Δ* and *gis1Δ* strains grown on SC (solid bars) and LN (striped bars) were assayed for β-galactosidase activity/reporter copy. **d** Wildtype diploid BY4743, *msn2Δ* and *msn4Δ* derivatives or wildtype haploid BY4741 and double-deletion derivative *msn2Δ msn4Δ* were grown on SC (solid bars) and LN (striped bars) were assayed for β-galactosidase activity/reporter copy. All strains exhibited significantly higher β-galactosidase activity in LN compared to the same strain in SC. In addition, both wildtype and *msn2Δ msn4Δ* BY4743 diploid exhibited higher activity than haploid cells. Where *rim15Δ* or *gis1Δ* strains showed a significant reduction in activity on both SC and LN media, neither *msn2Δ msn4Δ* single or double mutations decreased reporter gene activity. Error Bars = SEM, *n* = 3.*Compared to BY4743 cells in LN. #Significant compared to BY4743 in SC. (One-Way ANOVA, Tukey’s HSD, *p* ≤ 0.05, *n* = 3)

The Tor pathway is the master governor of cellular resources integrating the availability of nutrients and stress signaling (Fig. 5a). To examine the role of the Tor pathway on the polymerase switch, strains lacking Tor1 or the downstream kinase Sch9 were examined for reporter gene activity. Surprisingly, the absence of Tor1 did not significantly alter β-galactosidase activity in either abundant or limiting nitrogen conditions. However, the absence of downstream kinase Sch9 resulted in a significant increase in activity during abundant nitrogen, consistent with a role of inhibiting the polymerase switch. Although it is surprising that *tor1∆* and *sch9∆* strains result in differing reporter gene expression, Sch9 is also activated by Pkh1/2 of the Cell Wall Integrity and sphingolipid signaling pathways presenting an alternative activation mechanism (Fig. 5a) (Voordeckers et al. 2011; Deprez et al. 2018). The Rim15 kinase integrates signals from nitrogen sensing Tor pathway via Sch9, and carbon sensing PKA kinase. Phosphorylation by these nutrient-sensing pathways sequesters Rim15 in the cytoplasm inhibiting the activation of the downstream stress transcription factors Msn2, Msn4, Gis1, Rph1 and Hsf1 (Lee et al. 2013). Therefore, if the polymerase switch is regulated through Sch9-Rim15 signaling, the absence of Rim15, or downstream transcription factors would result in the absence or reduction reporter gene activity. To investigate this hypothesis, we examined *rim15∆* strains as well as deletion strains of its downstream targets *msn2∆* *msn4∆* and *gis1∆* (Fig. 5c). Absence of the Rim15 kinase or Gis1 similarly reduced both non-induced and induced reporter gene activity. We examined *msn2∆* and *msn4∆* in the diploid strain BY4743 and the *msn2∆* *msn4∆* double mutant in the haploid background BY4741 for β-galactosidase activity in either abundant or limiting nitrogen conditions (Fig. 5c). The wildtype haploid shows significantly higher reporter gene activity in both conditions compared to its diploid parent BY4743. Furthermore, the absence of Msn2 or Msn4 (BY4743) during nitrogen deprivation showed significantly higher reporter gene activity than wildtype, while the double-deletion strain *msn2∆* *msn4∆* showed no difference in activity in either condition. Although these results suggest that the regulation in diploids may be different than haploids, the absence of msn2 or msn4 does not decrease β-galactosidase activity, thereby eliminating them as possible activators of the polymerase switch.

The highly conserved, essential Heat Shock transcription factor Hsf1 is activated upon multiple stresses beyond heat shock including oxidative stress, osmotic stress, unfolded protein response, diauxic shift (Hahn et al. 2004) and errors in ribosome assembly (Albert et al. 2019). Hsf1 has putative binding sites within the rDNA pol II promoter and a 200 bp deletion encompassing the binding site results in no reporter gene activity (data not shown). Hsf1 is phosphorylated by Rim15 kinase (Lee et al. 2013) making it an attractive candidate for regulating pol II rRNA synthesis. Furthermore, following 12 h of nitrogen deprivation, Hsf1 target genes *HSP12, CTT1, HSP26* are highly upregulated with *HSP26* expression increasing fourfold (Gasch et al. 2000; Yamamoto et al. 2005).

**Fig. 6:**
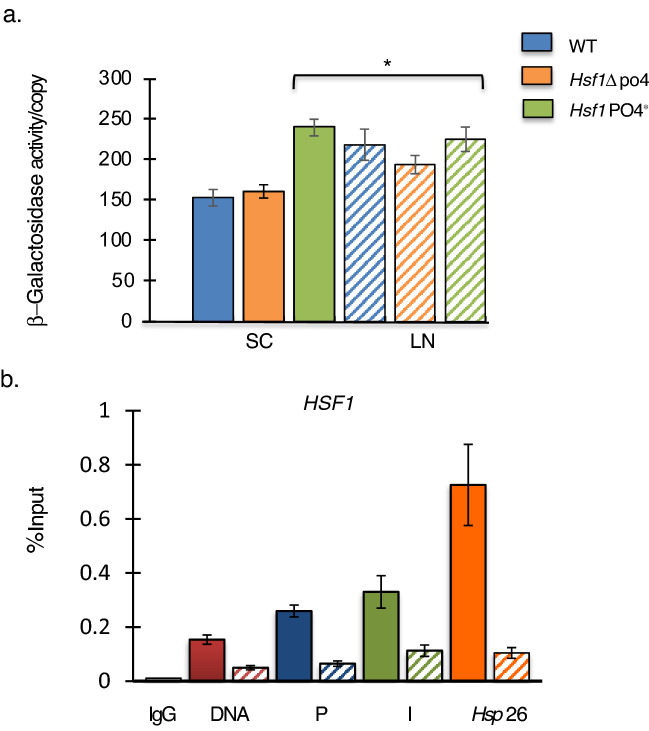
Hsf1 induces pol II rDNA synthesis. **a** W3031a wildtype, Hsf1^∆po4^ and Hsf1^PO4*^ derivatives grown in SC (solid bars) or in nitrogen deprivation medium LN (striped bars) were assayed for β-galactosidase activity/reporter copy. While Hsf1^∆po4^ cells showed wildtype activity in both SC and LN media, Hsf1^PO4*^ cells showed elevated β-galactosidase activity/reporter copy in either condition. Error Bars = SEM. *compared to W3031a WT cells in SC. (One-Way ANOVA, Tukey’s HSD *p* < 0.001, *n* = 3). **b** Wildtype BY4741 cells with the rDNA-T7 reporter gene integrated into a single rDNA repeat and pFES17 were grown in SC media (solid bar) prior to transfer to LN media for 12 h (striped bar). Harvested cells were subjected to ChIP using anti-Hsf1 antibody (gift of D. Gross) and the purified DNA was analyzed by q-PCR using the same primers as in Fig. 3a. Binding of Hsf1 in LN was significantly reduced compared to binding of Hsf1 to the same region from cells grown in SC. Binding to the Hsf1 target gene, *HSP26* promoter is shown as a control. Normal IgG antibody was used as negative controls for the experiment. Results are presented as % input. Error Bars = SEM. (Independent Student *T* test, *p* < 0.05, *n* = 3)

Hsf1 induction is regulated by two mechanisms; Hsp70 chaperones inhibit Hsf1 activation during non-stress conditions. Upon induction, Hsp70 chaperones are released activating Hsf1. Independently, phosphorylation by stress kinases modulate the level of I induction (Zheng et al. 2016). To investigate the role of the essential Hsf1factor, we examined the effect of two mutant alleles of *HSF1*. Although still regulated by Hsp70, the Hsf1^∆po4^ allele contains 152 serine/threonine to alanine substitutions rendering it unable to be phosphorylated. Thus, Hsf1^∆po4^ is still induced by heat shock, but to lower levels. The Hsf1^PO4*^ allele with 116 serine/threonine to aspartate substitutions mimics hyper-phosphorylation resulting in constitutive expression of Hsf1 target genes (Zheng et al. 2016) (Fig. 6a). In our experimental system, wildtype W3031a and Hsf1^∆po4^ behave similarly with low levels of β-galactosidase activity during abundant nitrogen and significantly elevated levels of activity following 12 h of nitrogen deprivation. In contrast, the constitutive Hsf1^PO4*^ allele showed induced levels of enzyme activity in both normal nitrogen and nitrogen deprivation conditions. Thus, phosphorylation of Hsf1 during non-stress conditions is sufficient to trigger the polymerase switch demonstrating that Hsf1 regulates of rRNA synthesis by pol II.

To confirm that Hsf1 directly activates the polymerase switch, we investigated the ability of Hsf1 to bind to the rDNA promoter by ChIP experiments. As expected, Hsf1 binds constitutively to both the *HSP26* promoter and rDNA promoters. Hsf1 binding to the *HSP26* promoter decreased sevenfold during nitrogen deprivation despite the increase in *HSP26* transcription (Fig. 6b). Likewise, Hsf1 binding to the rDNA promoter mimics its binding to the *HSP26* promoter; Hsf1 binds to the pol II rDNA promoter in all three contexts, the entire repeat, plasmid and single-tagged rDNA repeat. During nitrogen deprivation, Hsf1 rDNA binding is also reduced ~ threefold. Hsf1 binding to the single-tagged rDNA repeat is comparable to its binding to *HSP26* promoter during nitrogen deprivation. Thus, this decrease in Hsf1 binding to target promoters during chronic nitrogen deprivation must be a response to ongoing stress. ChIP experiments were also conducted to determine if Msn2, Msn4, or Gis1 bound to the pol II rDNA promoter in low nitrogen conditions, in all cases no significant binding of Gis1, Msn2 or Msn4 to the pol II rDNA promoter was detected. Overall, these data show that Hsf1 is activated during nitrogen deprivation via stress induction machinery to induce the polymerase switch.

## Discussion

Although initially discovered in response to mitochondrial dysfunction, this study demonstrates that nitrogen limitation also induces the polymerase switch. The polymerase switch utilizes the same components required for stress-induced transcription: stress signaling pathways, the mediator, Rpd3, and Hsf1. After 12 h growth in low nitrogen media, expression studies (Gasch et al. 2000) show that many of the highly expressed genes (expression above 2.0-fold) are those involved in response to nitrogen limitation: increased expression of the general amino acid permease gene *GAP1*, derepression of NCR genes (*DAL4, DAL 5, DAL 7, DAL 80, DUR3*), utilization of alternative N sources (*PUT1, DUR3*), as well as, nitrogen related autophagy genes (*ATG1, ATG 8, ATG34, ATG 39, ECM38*). However, expression of heat shock protein genes (*HSP12, HSP26, HSP42*, *HSP104*) and oxidative stress (*CTT1, CUP1, GTT1, GPX1*) and osmotic stress genes were also increased 2- or more-fold, providing evidence of ongoing oxidative stress, depletion of carbon sources and preparation for the diauxic shift. Since the pol II rDNA promoter has putative binding sites for transcription factors activated in these environments (Table 1), oxidative stress and additional nutrient limitation are likely contributing to the polymerase switch in our conditions. Indeed, preliminary experiments suggest that chronic oxidative stress and osmotic shock in the presence of abundant nutrients also trigger polymerase switch. Therefore, other nutrient depletion scenarios or chronic stresses may well trigger the polymerase switch independently as well as in concert with nitrogen deprivation.

In retrospect, it is reasonable that Hsf1 regulates the polymerase switch. The activation of Hsf1 during stress corresponds to the induction of the polymerase switch; both are regulated by the Sch9/Rim15 pathway as well as other signaling pathways (Zhang and Cao 2017). Both Hsf1 and the polymerase switch require the mediator and Rpd3 to induce stress response (Alejandro-Osorio et al. 2009; Kim and Gross 2013). Hsf1 shows significant binding to the rDNA promoter mimicking its binding to the Hsf1 target gene, *HSP26*, during nitrogen deprivation. What is puzzling is the decrease in Hsf1 binding to both the pol II rDNA promoter and the *HSP26* promoter following 12 h of nitrogen limitation. Based on the stress, Rim15, Yak1 and Mck1 kinases act coordinately to activate Msn2/4, Gis1 and Hsf1 to increase trehalose and glycogen accumulation and reduce ROS accumulation extending the Chronological Lifespan (CLS) (Cao et al. 2016; Zhang and Cao 2017). Therefore, it is highly likely that other stress transcription factors were activated in our experimental conditions. Alternatively, the role of Hsf1 may be similar to its role in Ribosomal Assembly Stress Reponse (RAStR) whereby unassembled ribosomal proteins aggregate resulting in the redistribution of HSP70 from Hsf1 to ribosomal protein aggregates leading to the induction of Hsf1 targets (Albert et al. 2019).

### A proposed model of Pol II rRNA synthesis during nitrogen deprivation

**Fig. 7:**
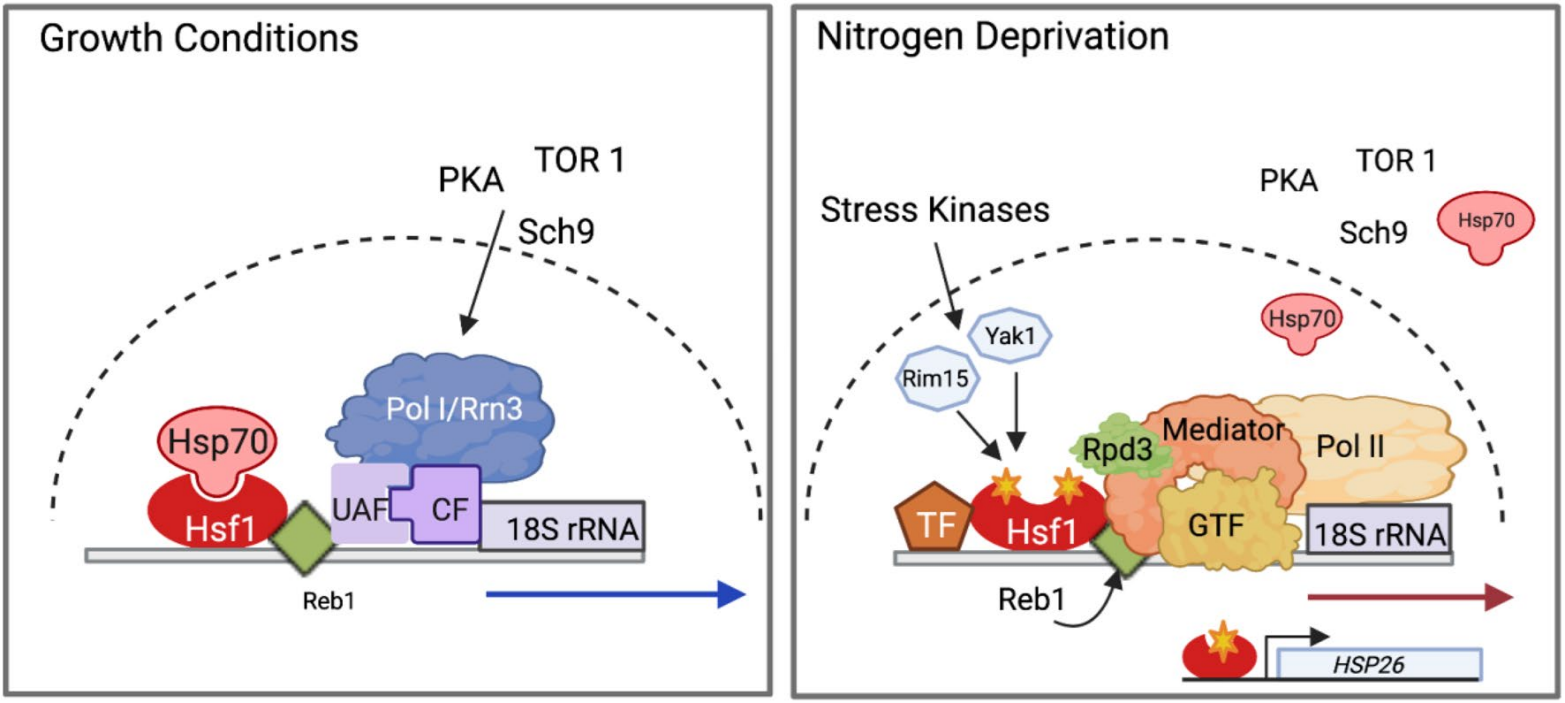
Model of the polymerase switch. **a** During nutrient rich conditions, TOR, PKA and Sch9 activate the formation of the pol I pre-initiation complex consisting of UAF, CF,TBP (not shown) and pol I/Rrn3. Hsf1 and its inhibitor HSP70, as well as Reb1 bind upstream of the pol I promoter. The pol I precursor rRNA is shown as a blue arrow. **b** In nitrogen deprivation conditions, TOR, PKA and Sch9 are inactive allowing the activation of Rim1 and Yak1 kinases and the subsequent phosphorylation (star) of Hsf1. Hsp70 is released allowing Hsf1 to recruit the mediator, Rpd3, and pol II transcription machinery and Pol II rRNA transcription (red arrow). Ongoing oxidative stress and nutrient depletion recruit Hsf1 to other stress promoters resulting in a decrease in Hsf1 binding at the pol II rDNA promoter. Other stress transcription factors (TF) may bind to the pol II rDNA promoter to maintain pol II rRNA transcription. The nuclear envelop is shown as a dashed line. Created with BioRender.com

During nutrient rich conditions, Tor/Sch9 and PKA activate pol I transcription (Fig. 7). Although Hsf1 is constitutively bound to the pol II rDNA promoter, it is inhibited by Hsp70. Following 12 h of nitrogen deprivation, limiting nitrogen in addition to reduction of glucose and increase in ROS (Broach 2012) results in the activation stress kinase Rim15 and Yak1 and/or Mck1. Inhibition of Hsf1 bound at the rDNA promoter by Hsp70 is relieved and phosphorylation of Hsf1 activates recruitment of Rpd3, mediator and general transcription machinery (Le Breton and Mayer 2016; Veri et al. 2018). In concert, activation of Gis, Uvs1, Yap 5 or other stress transcription factors may contribute to pol II rRNA synthesis. Some reduction in Hsf1 binding to the rDNA promoter occurs with it redistribution to other stress response promoters.

### Fine tuning of rRNA synthesis

During quiescence, stationary phase and or chronic environmental stress, rRNA synthesis rapidly decreases to < 10% maximal levels; however, rRNA and ribosomes continue to be synthesized (Warner 1999; Fahy et al. 2005; Davidson et al. 2011). Since pol II rRNA is assembled into functional ribosomes during mitochondrial dysfunction (Conrad-Webb and Butow 1995) or in the absence of UAF (Keys et al. 1996; Oakes et al. 1999; Vu et al. 1999; Siddiqi et al. 2001), pol II rRNA incorporation into ribosomes may allow the continued synthesis of stress-induced proteins facilitating survival.

A second role for the polymerase switch may be to fine-tune rRNA synthesis. In recent years, it has become apparent that long noncoding RNAs (lncRNAs) derived from the ribosomal repeat play a role in modulation of the rDNA chromatin, rDNA instability and rRNA synthesis. In yeast, pol II transcription from the Enhancer promoter (E-pro) results in rDNA instability. Furthermore, accumulation of these NTS1 transcripts in the absence of the CCR-Not1 complex is coupled not only with chromosomal instability, but with a reduction in rRNA synthesis and slow growth (Hosoyamada et al. 2019). In mammalian cells, heat shock, hypotonic conditions or serum deprivation results in the decrease of pol I transcription, while pol II transcription of the lncRNA PAPAS (promotor and pre-rRNA anti-sense) increases forming an anti-sense 35S RNA (Bierhoff et al. 2010). PAPAS then forms a triplex structure with the rDNA promoter (Zhao et al. 2016) where it recruits the histone methyl transferase Suv4-20h2 or Nucleosome Remodeling and Acetylation Complex (NuRD) altering the chromatin of the rDNA promoter to further inhibit pol I transcription (Bierhoff et al. 2014; Zhao et al. 2016). Zhao et al. (2018). Thus, PAPAS reinforces the reduction in pol I transcription.

Several lncRNAs in yeast, related to the 35S rRNA have been described that may play a similar role as PAPAS. The mitochondrial protein Tar1 is encoded within the 25S rRNA in the opposite orientation to the 35S rRNA (Coelho et al. 2002; Bonawitz et al. 2008) and is highly expressed during quiescence (Aragon et al. 2008). A second gene *RRT15* appears to have arisen from a duplication event of the upstream rDNA repeat and contains ~ 232 nucleotides of near identity to the 25S rRNA. Transcribed in the opposite orientation to the 25S rRNA, *RRT15* responds to similar regulators as the polymerase switch including Hsf1, Yap1 and the Mediator (Med4 and Sin4) (Hu et al. 2007; Venters et al. 2011; Cherry et al. 2012). Interestingly, *RRT15* is reported to enhance pol I transcription since a transposon insertion decreases levels of rDNA transcription (Hontz et al. 2009) suggesting that either the Rrt15 mRNA or protein may influence rRNA transcription. Mayan described a third pol II transcript complementary to the 35S 5’ ETS that binds Reb1 allowing the formation of a chromatin loop thought to enhancing pol I or pol III cycling and transcription (Mayan and Aragon 2010; Mayan 2013). Finally, YLR154A-G, YLR154C-G and XUT_12-F188 are a series of small transcripts overlapping the 35S rRNA, but their impact on rRNA synthesis, if any, has yet to be identified (Cherry et al. 2012). Accordingly, rDNA lncRNAs that overlap the large portions of the 35S transcription unit from promoter to terminator may act to modulate rRNA synthesis. Although the polymerase switch results in functional rRNA, pol II transcription may act to inhibit or maintain chromatin unfavorable to pol I.

The coordinated synthesis and assembly of ribosomes is essential. This study introduces a new development in ribosome synthesis during chronic stress -pol II synthesis of rRNA in response to prolonged nitrogen deprivation and perhaps other chronic stresses. It further demonstrates that activation of Hsf1 in concert with Rpd3 induces the polymerase switch linking for the first time Hsf1 and chronic stress with pol II synthesis of rRNA. However, Hsf1 has been linked to imbalance between ribosomal proteins and rRNAs or Ribosomal Assembly Stress Response (RAStR). This stress results in the downregulation of ribosomal protein genes and upregulation of Hsf1 targets as HSF70 is released from Hsf1 (Albert et al. 2019). Further investigation is needed to determine if the polymerase switch also occurs as a component of the RAStR and to dissect the contribution of Hsf1 and other stress transcription factors in the polymerase switch as well as in chronic stress. Whether pol II synthesis of rRNA plays a regulatory role further inhibiting pol I transcription or plays a stress adaption role providing rRNA for ribosome assembly is yet to be elucidated, and thus defines an important area for future research. This research also implicates the induction of a polymerase switch in response to chronic stress conditions as another important area for further research to understand the intricate regulation of rRNA and ribosome synthesis.

## References

[CR1] Albert B, Kos-Braun IC, Henras AK, Dez C, Rueda MP (2019). A ribosome assembly stress response regulates transcription to maintain proteome homeostasis. Elife.

[CR2] Alejandro-Osorio AL, Huebert DJ, Porcaro DT, Sonntag ME, Nillasithanukroh S (2009). The histone deacetylase Rpd3p is required for transient changes in genomic expression in response to stress. Genome Biol.

[CR3] Ansari SA, Morse RH (2012). Selective role of Mediator tail module in the transcription of highly regulated genes in yeast. Transcription.

[CR4] Ansari SA, Morse RH (2013). Mechanisms of mediator complex action in transcriptional activation. Cell Mol Life Sci.

[CR5] Aragon AD, Rodriguez AL, Meirelles O, Roy S, Davidson GS (2008). Characterization of differentiated quiescent and nonquiescent cells in yeast stationary-phase cultures. Mol Biol Cell.

[CR6] Baker LA, Ueberheide BM, Dewell S, Chait BT, Zheng D (2013). The yeast Snt2 protein coordinates the transcriptional response to hydrogen peroxide-mediated oxidative stress. Mol Cell Biol.

[CR7] Bierhoff H, Schmitz K, Maass F, Ye J, Grummt I (2010). Noncoding transcripts in sense and antisense orientation regulate the epigenetic state of ribosomal RNA genes. Cold Spring Harb Symp Quant Biol.

[CR8] Bierhoff H, Dammert MA, Brocks D, Dambacher S, Schotta G (2014). Quiescence-induced LncRNAs trigger H4K20 trimethylation and transcriptional silencing. Mol Cell.

[CR9] Bonawitz ND, Chatenay-Lapointe M, Wearn CM, Shadel GS (2008). Expression of the rDNA-encoded mitochondrial protein Tar1p is stringently controlled and responds differentially to mitochondrial respiratory demand and dysfunction. Curr Genet.

[CR10] Broach JR (2012). Nutritional control of growth and development in yeast. Genetics.

[CR11] Cao L, Tang Y, Quan Z, Zhang Z, Oliver SG (2016). Chronological lifespan in yeast is dependent on the accumulation of storage carbohydrates mediated by Yak1, Mck1 and Rim15 kinases. PLoS Genet.

[CR12] Chedin S, Laferte A, Hoang T, Lafontaine DL, Riva M (2007). Is ribosome synthesis controlled by pol I transcription?. Cell Cycle.

[CR13] Cherry JM, Hong EL, Amundsen C, Balakrishnan R, Binkley G (2012). *Saccharomyces* Genome Database: the genomics resource of budding yeast. Nucleic Acids Res.

[CR14] Coelho PSR, Bryan AC, Kumar A, Shadel GS, Snyder M (2002). A novel mitochondrial protein, Tar1p, is encoded on the antisense strand of the nuclear 25S rDNA. Genes Dev.

[CR15] Conrad-Webb H, Butow RA (1995). A polymerase switch in the synthesis of rRNA in *Saccharomyces**cerevisiae*. Mol Cell Biol.

[CR16] Davidson GS, Joe RM, Roy S, Meirelles O, Allen CP (2011). The proteomics of quiescent and nonquiescent cell differentiation in yeast stationary-phase cultures. Mol Biol Cell.

[CR17] de Boer CG, Hughes TR (2011). YeTFaSCo: a database of evaluated yeast transcription factor sequence specificities. Nucleic Acids Res.

[CR18] Deprez MA, Eskes E, Wilms T, Ludovico P, Winderickx J (2018). pH homeostasis links the nutrient sensing PKA/TORC1/Sch9 menage-a-trois to stress tolerance and longevity. Microb Cell.

[CR19] Dhar VN, Miller DA, Miller OJ (1985). Transcription of mouse rDNA and associated formation of the nucleolus organizer region after gene transfer and amplification in Chinese hamster cells. Mol Cell Biol.

[CR20] Dhar VN, Miller DA, Kulkarni AB, Miller OJ (1987). Human ribosomal DNA fragments amplified in hamster cells are transcribed only by RNA polymerase II and are not silver stained. Mol Cell Biol.

[CR21] Dilova I, Powers T (2006). Accounting for strain-specific differences during RTG target gene regulation in *Saccharomyces cerevisiae*. FEMS Yeast Res.

[CR22] Doelling JH, Pikaard CS (1996). Species-specificity of rRNA gene transcription in plants manifested as a switch in RNA polymerase specificity. Nucleic Acids Res.

[CR23] Fahy D, Conconi A, Smerdon MJ (2005). Rapid changes in transcription and chromatin structure of ribosomal genes in yeast during growth phase transitions. Exp Cell Res.

[CR24] Fan X, Chou DM, Struhl K (2006). Activator-specific recruitment of mediator in vivo. Nat Struct Mol Biol.

[CR25] Fath S, Milkereit P, Peyroche G, Riva M, Carles C (2001). Differential roles of phosphorylation in the formation of transcriptional active RNA polymerase I. Proc Natl Acad Sci USA.

[CR26] Gartenberg MR, Smith JS (2016). The nuts and bolts of transcriptionally silent chromatin in *Saccharomyces cerevisiae*. Genetics.

[CR27] Gasch AP, Spellman PT, Kao CM, Carmel-Harel O, Eisen MB (2000). Genomic expression programs in the response of yeast cells to environmental changes. Mol Biol Cell.

[CR28] Ghaemmaghami S, Huh WK, Bower K, Howson RW, Belle A (2003). Global analysis of protein expression in yeast. Nature.

[CR29] Gietz RD, Woods RA (2002). Transformation of yeast by lithium acetate/single-stranded carrier DNA/polyethylene glycol method. Methods Enzymol.

[CR30] Goetze H, Wittner M, Hamperl S, Hondele M, Merz K (2010). Alternative chromatin structures of the 35S rRNA genes in *Saccharomyces cerevisiae* provide a molecular basis for the selective recruitment of RNA polymerases I and II. Mol Cell Biol.

[CR31] Grummt I (2010). Wisely chosen paths–regulation of rRNA synthesis: delivered on 30 June 2010 at the 35th FEBS Congress in Gothenburg, Sweden. Febs J.

[CR32] Hahn JS, Hu Z, Thiele DJ, Iyer VR (2004). Genome-wide analysis of the biology of stress responses through heat shock transcription factor. Mol Cell Biol.

[CR33] Hontz RD, Niederer RO, Johnson JM, Smith JS (2009). Genetic identification of factors that modulate ribosomal DNA transcription in *Saccharomyces cerevisiae*. Genetics.

[CR34] Hosoyamada S, Sasaki M, Kobayashi T (2019). The CCR4-NOT complex maintains stability and transcription of rrna genes by repressing antisense transcripts. Mol Cell Biol.

[CR35] Hu Z, Killion PJ, Iyer VR (2007). Genetic reconstruction of a functional transcriptional regulatory network. Nat Genet.

[CR36] Keys DA, Lee BS, Dodd JA, Nguyen TT, Vu L (1996). Multiprotein transcription factor UAF interacts with the upstream element of the yeast RNA polymerase I promoter and forms a stable preinitiation complex. Genes Dev.

[CR37] Kief DR, Warner JR (1981). Coordinate control of syntheses of ribosomal ribonucleic acid and ribosomal proteins during nutritional shift-up in Saccharomyces cerevisiae. Mol Cell Biol.

[CR38] Kim S, Gross DS (2013). Mediator recruitment to heat shock genes requires dual Hsf1 activation domains and mediator tail subunits Med15 and Med16. J Biol Chem.

[CR39] Kurdistani SK, Grunstein M (2003). In vivo protein-protein and protein-DNA crosslinking for genomewide binding microarray. Methods.

[CR40] Laferté A, Favry E, Sentenac A, Riva M, Carles C (2006). The transcriptional activity of RNA polymerase I is a key determinant for the level of all ribosome components. Genes Dev.

[CR41] Le Breton L, Mayer MP (2016). A model for handling cell stress. Elife.

[CR42] Lee P, Kim MS, Paik SM, Choi SH, Cho BR (2013). Rim15-dependent activation of Hsf1 and Msn2/4 transcription factors by direct phosphorylation in *Saccharomyces cerevisiae*. FEBS Lett.

[CR43] Liu Z, Butow RA (1999). A transcriptional switch in the expression of yeast tricarboxylic acid cycle genes in response to a reduction or loss of respiratory function. Mol Cell Biol.

[CR44] Livak KJ, Schmittgen TD (2001). Analysis of relative gene expression data using real-time quantitative PCR and the 2(-Delta Delta C(T)) Method. Methods.

[CR45] Lopata MA, Cleveland DW, Sollner-Webb B (1986). RNA polymerase specificity of mRNA production and enhancer action. Proc Natl Acad Sci USA.

[CR46] Mayan MD (2013). RNAP-II transcribes two small RNAs at the promoter and terminator regions of the RNAP-I gene in *Saccharomyces cerevisiae*. Yeast.

[CR47] Mayan M, Aragon L (2010). Cis-interactions between non-coding ribosomal spacers dependent on RNAP-II separate RNAP-I and RNAP-III transcription domains. Cell Cycle.

[CR48] Merz K, Hondele M, Goetze H, Gmelch K, Stoeckl U (2008). Actively transcribed rRNA genes in *S. cerevisiae* are organized in a specialized chromatin associated with the high-mobility group protein Hmo1 and are largely devoid of histone molecules. Genes Dev.

[CR49] Morrow BE, Johnson SP, Warner JR (1989). Proteins that bind to the yeast rDNA enhancer. J Biol Chem.

[CR50] Moss T, Langlois F, Gagnon-Kugler T, Stefanovsky V (2007). A housekeeper with power of attorney: the rRNA genes in ribosome biogenesis. Cell Mol Life Sci.

[CR51] Myers AM, Tzagoloff A, Kinney DM, Lusty CJ (1986). Yeast shuttle and integrative vectors with multiple cloning sites suitable for construction of lacZ fusions. Gene.

[CR52] Oakes M, Siddiqi I, Vu L, Aris J, Nomura M (1999). Transcription factor UAF, expansion and contraction of ribosomal DNA (rDNA) repeats, and RNA polymerase switch in transcription of yeast rDNA. Mol Cell Biol.

[CR53] Oakes ML, Siddiqi I, French SL, Vu L, Sato M (2006). Role of histone deacetylase Rpd3 in regulating rRNA gene transcription and nucleolar structure in yeast. Mol Cell Biol.

[CR54] Parikh VS, Morgan MM, Scott R, Clements LS, Butow RA (1987). The mitochondrial genotype can influence nuclear gene expression in yeast. Science.

[CR55] Parikh VS, Conrad-Webb H, Docherty R, Butow RA (1989). Interaction between the yeast mitochondrial and nuclear genomes influences the abundance of novel transcripts derived from the spacer region of the nuclear ribosomal DNA repeat. Mol Cell Biol.

[CR56] Paul E, Zhu ZI, Landsman D, Morse RH (2015). Genome-wide association of mediator and RNA polymerase II in wild-type and mediator mutant yeast. Mol Cell Biol.

[CR57] Roche B, Arcangioli B, Martienssen R (2017). Transcriptional reprogramming in cellular quiescence. RNA Biol.

[CR58] Rose M, Botstein D (1983). Construction and use of gene fusions to lacZ (beta-galactosidase) that are expressed in yeast. Methods Enzymol.

[CR59] Sertil O, Vemula A, Salmon SL, Morse RH, Lowry CV (2007). Direct role for the Rpd3 complex in transcriptional induction of the anaerobic DAN/TIR genes in yeast. Mol Cell Biol.

[CR60] Siddiqi IN, Dodd JA, Vu L, Eliason K, Oakes ML (2001). Transcription of chromosomal rRNA genes by both RNA polymerase I and II in yeast uaf30 mutants lacking the 30 kDa subunit of transcription factor UAF. Embo J.

[CR61] Smale ST, Tjian R (1985). Transcription of herpes simplex virus tk sequences under the control of wild-type and mutant human RNA polymerase I promoters. Mol Cell Biol.

[CR62] Venters BJ, Wachi S, Mavrich TN, Andersen BE, Jena P (2011). A comprehensive genomic binding map of gene and chromatin regulatory proteins in *Saccharomyces*. Mol Cell.

[CR63] Veri AO, Robbins N, Cowen LE (2018). Regulation of the heat shock transcription factor Hsf1 in fungi: implications for temperature-dependent virulence traits. FEMS Yeast Res.

[CR64] Voordeckers K, Kimpe M, Haesendonckx S, Louwet W, Versele M (2011). Yeast 3-phosphoinositide-dependent protein kinase-1 (PDK1) orthologs Pkh1-3 differentially regulate phosphorylation of protein kinase A (PKA) and the protein kinase B (PKB)/S6K ortholog Sch9. J Biol Chem.

[CR65] Vu L, Siddiqi I, Lee BS, Josaitis CA, Nomura M (1999). RNA polymerase switch in transcription of yeast rDNA: role of transcription factor UAF (upstream activation factor) in silencing rDNA transcription by RNA polymerase II. Proc Natl Acad Sci USA.

[CR66] Warner JR (1999). The economics of ribosome biosynthesis in yeast. Trends Biochem Sci.

[CR67] Winzeler EA, Shoemaker DD, Astromoff A, Liang H, Anderson K (1999). Functional characterization of the *S. cerevisiae* genome by gene deletion and parallel analysis. Science.

[CR68] Woolford JL, Baserga SJ (2013). Ribosome biogenesis in the yeast *Saccharomyces cerevisiae*. Genetics.

[CR69] Worley J, Luo X, Capaldi AP (2013). Inositol pyrophosphates regulate cell growth and the environmental stress response by activating the HDAC Rpd3L. Cell Rep.

[CR70] Yamamoto A, Mizukami Y, Sakurai H (2005). Identification of a novel class of target genes and a novel type of binding sequence of heat shock transcription factor in Saccharomyces cerevisiae. J Biol Chem.

[CR71] Zhang N, Cao L (2017). Starvation signals in yeast are integrated to coordinate metabolic reprogramming and stress response to ensure longevity. Curr Genet.

[CR72] Zhao Z, Dammert MA, Hoppe S, Bierhoff H, Grummt I (2016). Heat shock represses rRNA synthesis by inactivation of TIF-IA and lncRNA-dependent changes in nucleosome positioning. Nucleic Acids Res.

[CR73] Zhao Z, Senturk N, Song C, Grummt I (2018). lncRNA PAPAS tethered to the rDNA enhancer recruits hypophosphorylated CHD4/NuRD to repress rRNA synthesis at elevated temperatures. Genes Dev.

[CR74] Zheng X, Krakowiak J, Patel N, Beyzavi A, Ezike J (2016). Dynamic control of Hsf1 during heat shock by a chaperone switch and phosphorylation. Elife.

